# Machine learning in image analysis in ophthalmology

**DOI:** 10.31744/einstein_journal/2021ED6860

**Published:** 2021-12-14

**Affiliations:** 1 Universidade de Coimbra Coimbra Portugal Universidade de Coimbra, Coimbra, Portugal.; 2 Universidade Federal de São Paulo São Paulo SP Brazil Universidade Federal de São Paulo, São Paulo, SP, Brazil.

Macular degeneration is the leading cause of irreversible blindness in developed countries in individuals aged over 50 years.^( 1 )^ Aiming to better diagnose and monitor the disease, algorithms have been developed to detect lesions in optical coherence tomography (OCT). The ability of machine learning algorithms to detect OCT lesions may already be comparable to that of retina specialists.^( 2 )^

The theory of machine learning consists of simulating tiny synapses of a human brain. Neural networks were inspired by human synapses and are mathematical models applied in pattern classification and recognition.

As in human learning, computers must be exposed to data to learn through examples. Neural networks enable this learning and the application of knowledge in the classification of unknown images. The main features taught to the computer for image analysis are colors, shapes, location, and contrast. The neural network is trained to activate different outputs for various images presented during training. After each image presented, an internal weight is provided, which strengthens certain “synaptic connections”.

The training process includes presenting images that are randomly separated into three groups: training, validation, and verification. The training group is used to adjust the weight of connected networks. The validation group is used to determine the best moment to finish training, and the verification group is subsequently applied, defining the performance of the algorithm.^( 3 )^

Studies on this new interpretation of computational patterns can improve the understanding of diseases, besides increasing the confidence of physicians in the diagnosis aided by machine learning techniques.

A study developed by Xu et al., required 654 spectral domain OCT images of patients with macular degeneration to have 96% accuracy in the identification of intraretinal fluids.^( 4 )^ Another study, developed by Chakravarthy et al., required 155 spectral domain OCT images to achieve 93% accuracy in the identification of intraretinal fluids.^( 5 )^ The study by Kermany et al., required 207,130 OCT images to have 96.6% accuracy in the identification of drusen.^( 6 )^ The study by Khalid et al., required 6,800 OCT images to achieve 98% accuracy in the detection of drusen^( 7 )^ ( Table 1 ).

**Table 1:** Number of images needed to teach the algorithms how to automatically detect lesions in optical coherence tomography

Study	Image	Accuracy (%)
Xu et al.^( 4 )^	Intraretinal fluids (654)	96
Chakravarthy et al.^( 5 )^	Intraretinal fluids (155)	93
Kermany et al.^( 6 )^	Drusen (207,130)	96.6
Khalid et al.^( 7 )^	Drusen (6,800)	98

It is therefore understood that the algorithms required more images of drusen than of intraretinal fluid to learn the pattern. This can possibly be explained by the size, location, and contrast difference between these two groups of lesions studied in OCT images. The recognition of machine standards involves attribution techniques with as little human intervention as possible.^( 3 )^

Computer vision uses pattern recognition. The classification model is usually based on the availability of a set of patterns that were used in the group of training images. The algorithm learning methodology occurs with the determination of random weights for learning, using the characteristics of the objects employed in the training set. The model adjusts the weights to get a correct image rating. The weight interaction is adapted according to the principle “punishment/reward”.^( 8 )^ This method is used in humans from birth to recognize the objects that surround us. This learning capacity has been developed over thousands of years of evolution, and has allowed humans to recognize food and predators appropriately. In the process of image recognition by the computer, an initial image segmentation occurs and, later, the extraction of the characteristics to be analyzed. In image segmentation, the object to be recognized is isolated from the rest of the image, and during the extraction of the characteristics, attribute vectors are assigned, decreasing the amount of information to classify it.^( 9 )^ It is interesting to be able to learn from algorithms how to identify patterns that are not naturally valued by humans.

As an example of the image segmentation methodology, there is the methodology of segmenting grayscale thresholds, used to establish the limits of the image.^( 9 )^ After the image segmentation process, algorithms begin to extract relevant resources to decrease the computational power required during the classification process. This information embedded into the process of developing vector attributes, provides the development of algorithms with less computational power to learn how to classify images.

Algorithms require a large number of images from which to be learned, requiring data from different populations, which creates a current problem in training algorithms for detecting rare diseases.^( 10 )^

**Figure 1:**
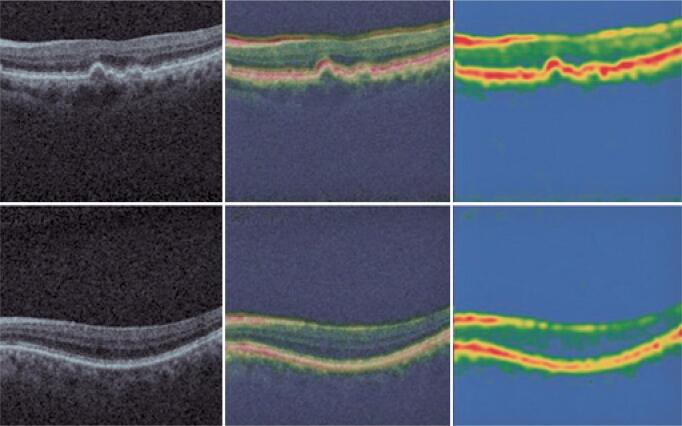
Heat maps that indicate how important is each image location for the algorithm classification

In the unsupervised learning process, in which there is no teacher to determine whether the output response is satisfactory, it is possible to learn from algorithms, improving understanding of how the weights they provide for certain decisions are different from humans. This may be assessed by heat maps that indicate how important each image location for the algorithm classification. This technique enables visualizing the parts of the image that are most important for the classification by the deep neural network. This provides further confirmation that the algorithm is, in fact, identifying the area of the photo that is important for diagnosis ( Figure 1 ).

Advances in the development of algorithms for image analyses have therefore proven promising in many areas of medicine, such as ophthalmology.
