# Supplementary material for: Machine learning in image analysis in ophthalmology
Source: Einstein (Sao Paulo). 2021 Dec 14;19:eED6860. doi: 10.31744/einstein_journal/2021ED6860 (PMC8687641; doi:10.31744/einstein_journal/2021ED6860)
Supplement: Translation

# Translation: Aprendizagem de máquina em análise de imagens na oftalmologia

**Affiliations:** 1 Coimbra Portugal Universidade de Coimbra, Coimbra, Portugal.; 2 São Paulo SP Brasil Universidade Federal de São Paulo, São Paulo, SP, Brasil.

A degeneração macular é a principal causa de cegueira irreversível em indivíduos com mais de 50 anos em países desenvolvidos.^( 1 )^Visando à melhor forma de diagnosticar e monitorar essa doença, foram desenvolvidos algoritmos para detecção de lesões de tomografia de coerência óptica (OCT - *optical coherence tomography* ). A capacidade dos algoritmos de aprendizado de máquina para detectar lesões de OCT já pode ser comparável a de especialistas em retina.^( 2 )^

A teoria do aprendizado da máquina consiste em simular pequenas sinapses de um cérebro humano. As redes neurais foram inspiradas nas sinapses humanas e são modelos matemáticos aplicados na classificação e no reconhecimento de padrões.

Assim como no aprendizado humano, os computadores devem ser expostos a dados para aprender por meio de exemplos. As redes neurais permitem esse aprendizado e a aplicação dos conhecimentos na classificação de imagens desconhecidas. Os principais fatores que são ensinados ao computador para análise de imagens são cores, formas, localização e contraste. A rede neural é treinada para ativar diferentes saídas para variadas imagens apresentadas durante o treinamento. Após cada imagem apresentada, é fornecido um peso interno, que fortalece certas “conexões sinápticas”.

O processo de treinamento inclui a apresentação de imagens que são divididas aleatoriamente em três grupos: treinamento, validação e verificação. O grupo de treinamento é usado para ajustar o peso das redes conectadas. O grupo de validação é utilizado para determinar o melhor momento para finalizar o treinamento, e, então, o grupo de verificação é aplicado, definindo o desempenho do algoritmo.^( 3 )^

Estudos sobre essa nova interpretação de padrões computacionais podem melhorar o entendimento das patologias, além de aumentar a confiança da classe médica no diagnóstico auxiliado por técnicas de aprendizado de máquina.

Estudo desenvolvido por Xu et al., necessitou de 654 imagens de OCT de domínio espectral de pacientes com degeneração macular para ter precisão de 96% na identificação de fluidos intrarretinais.^( 4 )^Outro estudo, este desenvolvido por Chakravarthy et al., necessitou de 155 imagens de OCT de domínio spectral para ter precisão de 93% na identificação de fluidos intrarretinianos.^( 5 )^O estudo de Kermany et al., precisou de 207.130 imagens de OCT para ter 96,6% de acurácia na identificação de drusas.^( 6 )^Já o estudo de Khalid et al., necessitou de 6.800 imagens de OCT para ter 98% de precisão na detecção de drusas^( 7 )^( Tabela 1 ).

**Tabela 1:** Número de imagens necessárias para ensinar aos algoritmos como detectar lesões automaticamente em exames de tomografia de coerência óptica

Estudo	Imagem	Acurácia (%)
Xu et al.^( 4 )^	Líquido intrarretiniano (654)	96
Chakravarthy et al.^( 5 )^	Líquido intrarretiniano (155)	93
Kermany et al.^( 6 )^	Drusas (207.130)	96,6
Khalid et al.^( 7 )^	Drusas (6.800)	98

Assim, entende-se que os algoritmos exigiram mais imagens de drusas do que de líquido intrarretiniano para aprender o padrão. Isso possivelmente pode ser explicado pelo tamanho, pela localização e pela diferença de contraste entre esses dois grupos de lesões estudadas nas imagens de OCT. O reconhecimento de padrões de máquina envolve técnicas de atribuição com o mínimo de intervenção humana possível.^( 3 )^

A visão computacional usa o reconhecimento de padrões. O modelo de classificação geralmente é baseado na disponibilidade de um conjunto de padrões que foram usados no grupo de imagens de treinamento. A metodologia de aprendizagem do algoritmo ocorre com a determinação de pesos aleatórios para a aprendizagem, utilizando as características dos objetos empregados no conjunto de treinamento. O modelo ajusta os pesos para obter uma classificação correta da imagem. A interação dos pesos é adaptada de acordo com o princípio “punição-recompensa”.^( 8 )^Esse método é usado em seres humanos desde o nascimento, para reconhecer os objetos que nos rodeiam. Essa capacidade de aprendizado foi desenvolvida ao longo de milhares de anos de evolução, e permitiu aos humanos reconhecerem alimentos e predadores de maneira adequada. No processo de reconhecimento da imagem pelo computador ocorre uma segmentação inicial da imagem e, posteriormente, a extração das características a serem analisadas. Na segmentação da imagem, o objeto a ser reconhecido é isolado do restante da imagem, e, durante a extração das características, são atribuídos vetores de atributos, diminuindo a quantidade de informações para classificá-la.^( 9 )^É interessante poder aprender com os algoritmos como identificar padrões que não são valorizados naturalmente pelos humanos.

Como exemplo da metodologia de segmentação de imagens, tem-se a metodologia de segmentação de limiares de tons de cinza para estabelecer os limites da imagem.^( 9 )^Após o processo de segmentação da imagem, os algoritmos começam a extrair recursos relevantes para diminuir o poder computacional necessário durante o processo de classificação. Essas informações, incorporadas ao processo de desenvolvimento de atributos vetoriais, proporcionam o desenvolvimento de algoritmos com menor poder computacional para aprender a classificar imagens.

Os algoritmos requerem um grande número de imagens para ser aprendidas, exigindo dados de diferentes populações, o que gera um problema atual no treinamento de algoritmos para detecção de doenças raras.^( 10 )^

No processo de aprendizagem não supervisionado, no qual não há professor para determinar se a resposta de saída é satisfatória, é possível aprender com os algoritmos, melhorando o entendimento sobre como os pesos que eles fornecem para certas decisões são diferentes dos humanos. Isso pode ser avaliado por mapas de calor que indicam o quão importante cada localização da imagem é fundamental para a classificação do algoritmo. Essa técnica permite visualizar as partes da imagem que são mais importantes na classificação da rede neural profunda. Isso fornece mais uma confirmação de que o algoritmo está, de fato, identificando a área da foto que é importante para o diagnóstico ( Figura 1 ).

**Figura 1:** Mapas de calor indicam o quão importante cada localização da imagem é fundamental para a classificação do algoritmo

Dessa forma, o avanço no desenvolvimento de algoritmos de análise de imagens tem se demonstrador promissor em muitas áreas de medicina, como a oftalmologia.
